# Evaluation of Perfusion Change According to Pancreatic Cancer and Pancreatic Duct Dilatation Using Free-Breathing Golden-Angle Radial Sparse Parallel (GRASP) Magnetic Resonance Imaging

**DOI:** 10.3390/diagnostics13040731

**Published:** 2023-02-15

**Authors:** Moonhyung Choi, Seungbae Yoon, Youngjoon Lee, Dongyeob Han

**Affiliations:** 1 Department of Radiology, Eunpyeong St. Mary’s Hospital, College of Medicine, The Catholic University of Korea, Seoul 03312, Republic of Korea; 2 Department of Internal Medicine, Eunpyeong St. Mary’s Hospital, College of Medicine, The Catholic University of Korea, Seoul 03312, Republic of Korea; 3 Siemens Healthineers Ltd., Seoul 06620, Republic of Korea

**Keywords:** perfusion imaging, magnetic resonance imaging, pancreas, pancreatic neoplasms

## Abstract

**Simple Summary:**

Pancreas MRI is used to differentiate pancreas focal lesions by evaluating the enhancement pattern of the focal lesion compared with the adjacent pancreatic parenchyma. Morphologic changes in the pancreas are common as a result of pancreatic cancer and may cause changes in the blood supply (perfusion) to the pancreas. A dynamic contrast-enhanced MRI (DCE-MRI) technique that can obtain MRI images in very short intervals of time (about 2–5 s) was developed, even for the organs that continuously move as a result of breathing. In the present study, we confirm a perfusion change in the pancreas with pancreatic cancer using DCE-MRI, with a longer peak-enhancement time, longer delay time, and higher peak concentration. We also present the correlations between perfusion and morphological changes in the pancreas. DCE-MRI may be helpful to understand the microscopic changes in the pancreatic parenchyma when a disease occurs.

**Abstract:**

Purpose: To evaluate perfusion changes in the pancreas with pancreatic cancer and pancreatic duct dilatation using dynamic contrast-enhanced MRI (DCE-MRI). Method: We evaluate the pancreas DCE-MRI of 75 patients. The qualitative analysis includes pancreas edge sharpness, motion artifacts, streak artifacts, noise, and overall image quality. The quantitative analysis includes measuring the pancreatic duct diameter and drawing six regions of interest (ROIs) in the three areas of the pancreas (head, body, and tail) and three vessels (aorta, celiac axis, and superior mesenteric artery) to measure the peak-enhancement time, delay time, and peak concentration. We evaluate the differences in three quantitative parameters among the ROIs and between patients with and without pancreatic cancer. The correlations between pancreatic duct diameter and delay time are also analyzed. Results: The pancreas DCE-MRI demonstrates good image quality, and respiratory motion artifacts show the highest score. The peak-enhancement time does not differ among the three vessels or among the three pancreas areas. The peak-enhancement time and concentrations in the pancreas body and tail and the delay time in the three pancreas areas are significantly longer (*p* < 0.05) in patients with pancreatic cancer than in those without pancreatic cancer. The delay time was significantly correlated with the pancreatic duct diameters in the head (*p* < 0.02) and body (*p* < 0.001). Conclusion: DCE-MRI can display the perfusion change in the pancreas with pancreatic cancer. A perfusion parameter in the pancreas is correlated with the pancreatic duct diameter reflecting a morphological change in the pancreas.

## 1. Introduction

Pancreatic ductal adenocarcinoma (PDAC), which is commonly known as pancreatic cancer, is one of the most devastating cancers and has a poor prognosis. Imaging findings of pancreatic cancer include hypodense/hypointense pancreatic focal lesions with duct dilatation and parenchymal atrophy [1,2]. Imaging findings for missed cases of pancreatic cancer include isoattenuation on CT or no contour deformation, although pancreatic duct dilatation is noted in 88% of patients [3]. Additionally, in most missed cases of pancreatic cancer, focal lesions are observed upon prediagnostic MRI examinations [4]. Prominent differences in the density/signal intensity between pancreatic parenchyma and pancreatic focal lesions may be important for detecting the latter. However, approximately 30% of CT or MRI examinations present suboptimal contrast dosing [3].

Contrast-enhanced pancreas MRI may be acquired using fixed time delays, bolus tracking, or a test bolus [5]. As the images were obtained at a predetermined time delay after contrast injection, the fixed time delay method cannot reflect the perfusion characteristics of each patient or organ. Because various factors can affect the arrival time of contrast material in the aorta and pancreas, bolus tracking or a test bolus is expected to be superior to fixed time delays. However, even bolus tracking cannot guarantee the optimal scan time for the pancreas. Dynamic contrast-enhanced imaging (DCE) of MRI (DCE-MRI) is a method used to evaluate organ perfusion or vascularity because dozens of images are obtained in one section at 2–4 s intervals. Therefore, it is possible to know when the contrast enhancement is at its peak and how high the contrast enhancement actually is in the tissue. It is difficult to obtain a DCE-MRI for the abdominal organs because they move continuously as a result of breathing. Several studies have investigated DCE-MRI using the test bolus technique, in which a proportion of the contrast media is used to evaluate the enhancement time in the pancreas [6,7]. The DCE-MRI enhancement pattern and parameters are useful for differentiating the pancreatic focal lesions [7,8,9,10]. DCE-MRI can also be used to evaluate vascularity in pancreatic cancer to predict responses to chemotherapy [6,11,12].

A golden-angle radial sparse parallel MRI (GRASP) that uses compressed sensing and parallel imaging to accelerate data acquisition was used, enabling to acquire DCE-MRI with high temporal and spatial resolutions [13,14]. Furthermore, continuously acquiring radial data with the angle of the radial lines raised by 111.25 degrees permits free-breathing continuous data acquisition, which is a significant benefit in abdominal imaging, where breathing motion artifacts are a problem. Although GRASP has been used to evaluate focal pancreatic lesions or focal hepatic lesions in previous research [11,15], studies have not focused on the perfusion characteristics of the pancreas. The DCE-MRI of the pancreas allows for the evaluation of both the perfusion and morphologic characteristics, which may be associated with each other. We thought that we could understand the contrast enhancement characteristics of the pancreas, which was a reference to interpret the enhancement pattern of the pancreatic focal lesions in patients with pancreatic duct dilatations. The purpose of the present study is to evaluate the perfusion changes in the pancreas with pancreatic cancer and pancreatic duct dilatation using DCE-MRI.

## 2. Materials and Methods

The institutional review board of our hospital approved this retrospective study (No. PC20RISI0057) and waived informed consent owing to the retrospective study design.

### 2.1. Subjects

Patients underwent an MRI for the pancreas to evaluate pancreas focal lesions, pancreatic duct dilatations, and gallbladder disease between April 2019 and January 2020 (n = 109). We excluded patients who underwent a non-enhanced MRI (n = 33). A patient who did not have a normal pancreatic parenchyma owing to severe atrophy was excluded. We did not exclude patients based on their final diagnosis Finally, a total of 75 patients (31 males, 44 females; mean age ± standard deviation, 71.5 ± 12.6 years old) were included, regardless of the presence of focal lesions. Patient characteristics, including available biopsy results, were collected from the electronic medical records.

### 2.2. MR Protocol

All of the MRI examinations were performed using 3T MRI systems (Magnetom Vida, Siemens Healthineers, Erlangen, Germany) with a 30-channel surface coil and 32- or 72-channel spine coil. Breath-hold half-Fourier acquisition single-shot turbo spin echo (HASTE) heavily T2-weighted imaging was obtained to evaluate the structures containing fluid such as the pancreatic duct and cyst, respiratory-triggered fast spin echo T2WI with fat suppression was obtained to evaluate the overall anatomical structure, and 3D gradient echo volumetric interpolated breath-hold examination (VIBE) T1-weighted imaging (T1WI) based on in- and opposed-phase was obtained to detect the fat component in the lesion, while diffusion-weighted imaging (DWI) with b values of 0, 50, 400, 800, and 1000 s/mm^2^ was obtained to acquire information about the cellularity or nature of tissue inside the lesion. For the DCE-MRI, 0.1 mmol/kg of gadoterate meglumine (Dotarem, Guerbet, Paris, France) was injected at a rate of 1.5 mL/s using a power injector, followed by a 20 mL saline flush. DCE-MRI was acquired using free-breathing golden-angle radial sparse parallel (GRASP) imaging. The temporal resolution was 13.5 s for the first 24 s, and then 8.4 s for 180 s and 13.5 s for the last 121 s. After performing the DCE-MRI, we also performed delayed-phase coronal and sagittal contrast-enhanced T1WI with a higher spatial resolution than DCE_MRI to obtain additional information about the lesion or anatomical structure. The detailed MR parameters are summarized in Table 1 and most of the sequences were performed with the commonly used MRI technique.

### 2.3. Image Analysis

Two abdominal radiologists with 24 and 12 years of experience independently evaluated the DCE-MRI image quality. They reviewed the images using cine mode, with all images from the same section being presented sequentially. Five items (pancreas edge sharpness, motion artifact, streak artifact, noise, and overall image quality) were evaluated with a five-point scale. A score of 1 indicated the most severe image degradation or the worst image quality, and a score of 5 indicated the fewest artifacts/least noise or the best image quality. Pancreas edge sharpness was evaluated on the section that covered the largest area of the pancreas. For the determination of the motion artifact, the presence of multiple lines parallel to the abdominal wall that caused the blurring of the abdominal wall was evaluated. Streak artifact usually appeared as multiple radial lines around the very bright structures or structures outside the field of view. Two radiologists reviewed all of the available images in consensus to determine whether a focal lesion was in the pancreas and made a diagnosis for the focal lesion.

**Figure 1:**
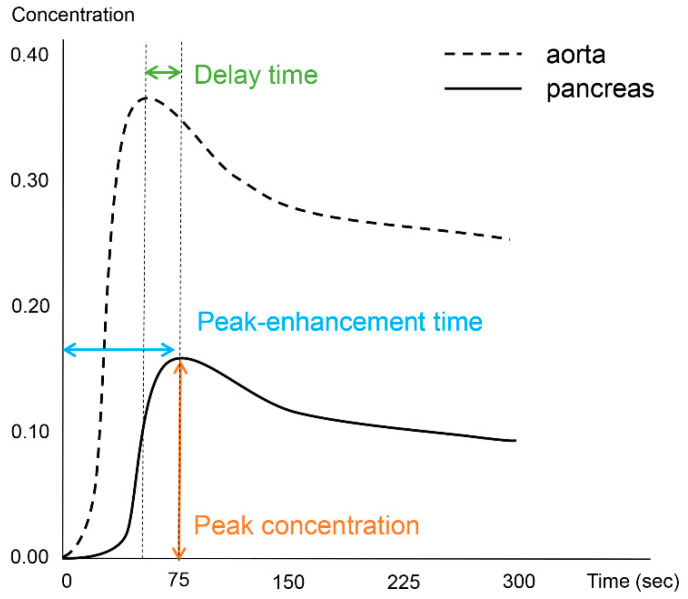
Schematic diagram illustrative of the investigated perfusion parameters.

Image processing and analysis for the DCE-MRI was performed using an application for evaluating DCE-MRI (MR Tissue4D) based on commercial software (Syngo.via VB30A, Siemens Healthineers). The perfusion maps were generated using a population-based arterial input function within a sphere-shaped volume of interest containing the entire pancreas and adjacent vessels. Although the software application provided parametric maps based on the Tofts model, we only used measurements from the time–intensity curve. One abdominal radiologist with 12 years of experience in pancreatic MRI performed the image analysis and measured the pancreatic duct diameter in the head, body, and tail of the pancreas. The radiologist also drew six regions of interest (ROIs) in three areas of the vessels (the descending aorta at the left crus level, celiac axis, and superior mesenteric artery (SMA)) and three areas of the pancreas (head, body, and tail). The demarcation of the head, body, and tail of the pancreas was based on the 8th edition of the American Joint Committee on Cancer (AJCC) system [16], as follows: the head is to the right of the superior mesenteric–portal vein confluence, the body is between the left border of the superior mesenteric vein and the left border of the aorta, and the tail is between the left border of the aorta and the hilum of the spleen. ROIs in the vessels were free-hand drawn as large as possible while avoiding the vessel wall, and those in the pancreas had sizes larger than 50 mm^2^. From each ROI, we measured the peak-enhancement time, which is the time between the start of image acquisition and the highest signal intensity. The delay time was based on the time elapsed between the peak-enhancement time in the aorta and the pancreas. The peak concentration in the ROI was recorded from the time–intensity curve. Figure 1 depicts the concepts of the investigated parameters (Figure 1).

### 2.4. Statistical Analysis

The results of the image quality analysis are presented as the mean and standard deviation, and inter-reader agreement between the two radiologists was analyzed using the intraclass correlation coefficient (ICC) with a two-way random effect model. The results were interpreted as follows: <0.50, poor agreement; 0.50–0.74, moderate agreement; 0.75–0.89, good agreement; and 0.90–1.00, excellent agreement. The peak-enhancement time and peak concentration in ROIs were compared among the vessels and pancreatic parenchyma using paired *t*-tests. The differences in the peak-enhancement time, delay time, and peak concentration between patients with and without pancreatic cancer and between patients with pancreatic cancer and other focal pancreatic lesions were analyzed using independent *t*-tests. A multivariate linear regression analysis was performed to identify the perfusion parameters related to pancreatic duct diameter in the pancreas head, body, and tail, respectively. The correlations between the pancreatic duct diameter and significant perfusion parameters were evaluated using the Pearson correlation coefficient (r). A value of *p* < 0.05 was considered statistically significant. Statistical analyses were performed in SPSS version 23.0 (IBM, Armonk, NY, USA).

## 3. Results

Among the 75 patients, 38 patients had a focal lesion in the pancreas. Pancreatic ductal adenocarcinoma, generally called pancreatic cancer, was radiologically diagnosed in 22 patients. Among them, 18 patients with available histological results were diagnosed with pancreatic ductal adenocarcinoma. The patients’ characteristics are summarized in Table 2.

A DCE-MRI of the pancreas with GRASP demonstrated an acceptable or good image quality (Table 3). The two radiologists commonly gave the highest mean score to respiratory motion artifacts. The mean scores of the overall image quality were 4.13 and 3.94 by readers 1 and 2, respectively. All of the items except the respiratory motion artifact (ICC, 0.462) showed a moderate or good inter-reader agreement, with an ICC higher than 0.650.

Although the peak-enhancement time in the aorta, SMA, and celiac axis was sequentially longer, significant differences were not observed among them (Table 4). The peak concentration was significantly lower in the celiac axis than in the aorta and SMA. Regional differences in the peak concentration were not noted in the pancreas.

**Figure 2:**
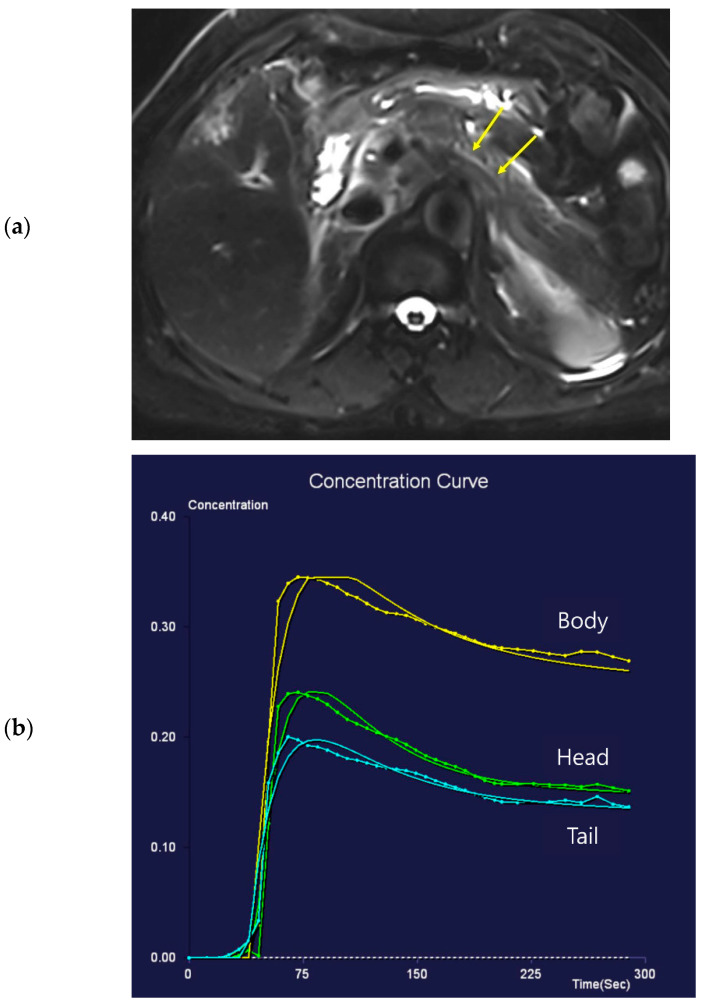
Seventy-one-year-old female patient undergoing an MRI to evaluate the gall bladder. There is no pancreatic dilatation (arrows) on the T2-weighted imaging (**a**) and no pancreatic focal lesion. Dynamic contrast-enhanced curves show rapid peak-enhancement and a gradual decrease in enhancement in the head, body, and tail (**b**).

**Figure 3:**
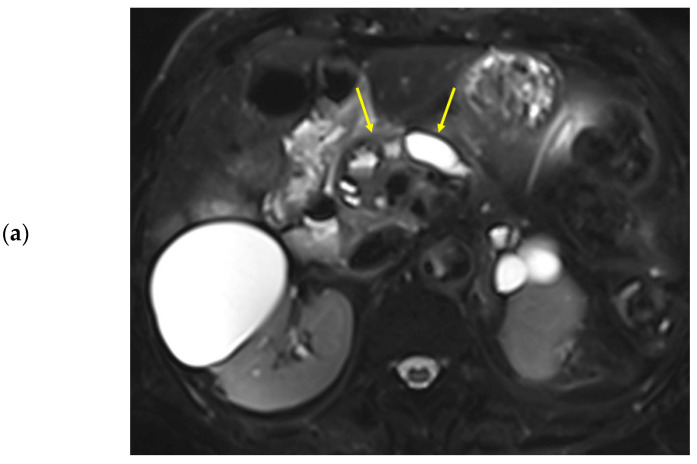

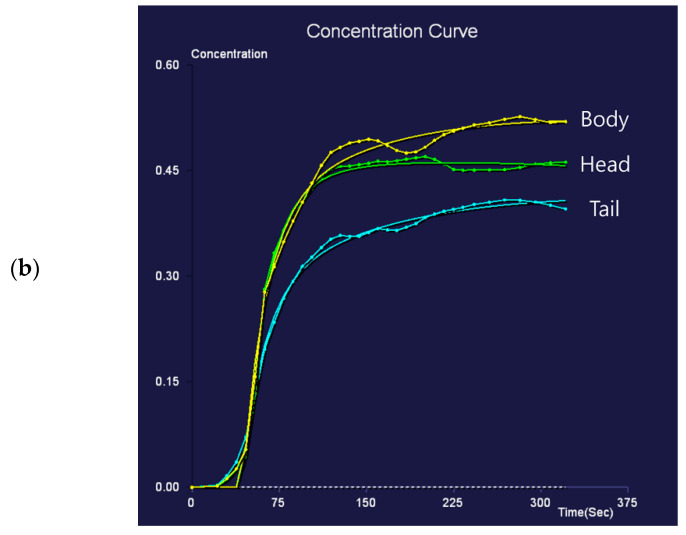
Eighty-nine-year-old female patient undergoing an MRI to evaluate pancreatic focal lesions. Diffuse pancreatic dilatation (arrows) is noted on T2-weighted imaging (**a**), and a 2.5 cm mass suspected to be pancreatic cancer is noted in the pancreatic head (not presented). Dynamic contrast-enhanced curves show gradual enhancement with stable signal intensity in the pancreas head, body, and tail (**b**).

The peak-enhancement time and peak concentration in the vessels did not differ because of the presence of pancreatic cancer (*p* > 0.05). The peak-enhancement time in the pancreatic body and tail and the delay time in the three areas of the pancreas were significantly longer in patients with pancreatic cancer (Table 5). The peak concentrations in the three pancreas areas were higher in patients with pancreatic cancer, although statistically significant differences were observed in the body and tail. Figure 2 and Figure 3 show two representative cases with and without pancreatic cancer. Among the patients with pancreatic focal lesions, the pancreatic duct diameter in the tail was significantly larger in patients with pancreatic cancer than in patients with other focal lesions, such as cyst or neuroendocrine tumor (diameter: 2.53 ± 1.64 mm versus 4.05 ± 2.23 mm, *p* = 0.037). Three perfusion parameters were shorter with benign lesions than with pancreatic cancer, and a clinical significance was noted in the tail (Table 5).

**Figure 4:**
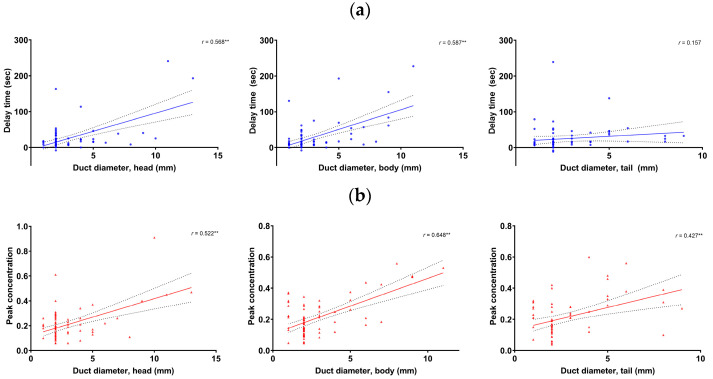
Correlation between the pancreatic duct diameter and the delay time (**a**) and peak concentration (**b**) in the pancreas head (**left**), body (**mid**), and tail (**right**), respectively. **: *p* < 0.001.

Multivariate regression analyses showed that the delay time and peak concentration in the pancreas head and body, as well as peak concentration in the tail, were significant factors related to the pancreatic duct diameter. The significant factors did not have multicollinearity. The pancreatic duct diameter was significantly correlated with the delay time in the head (r = 0.568, *p* < 0.001) and body (r = 0.587, *p* < 0.001), but not in the tail (*p* > 0.05). The pancreatic duct diameter was significantly correlated with the peak concentration in the head (r = 0.522, *p* < 0.001), body (r = 0.648, *p* < 0.001), and tail (r = 0.427, *p* < 0.001) (Figure 4).

## 4. Discussion

In this study, the DCE-MRI of the pancreas using GRASP provided an acceptable image quality. Pancreatic perfusion was changed by the presence of pancreatic cancer, and the peak-enhancement time and peak concentration were greater in patients with pancreatic cancer than in those without it. We also showed that the pancreatic parenchymal peak-enhancement time and delay time were prolonged with pancreatic duct dilatation in the pancreas head and body. Therefore, obtaining images with fixed time delays may not be appropriate for achieving the maximal enhancement of the pancreas in patients with pancreatic duct dilatation, which is the morphological change in pancreatic cancer. Considering our results, the DCE-MRI with continuous image acquisition at different time points may be useful for obtaining more information about perfusion in the pancreas and beneficial for acquiring images at the appropriate scan time.

The DCE-MRI of the pancreas was performed using GRASP. Acquiring the DCE-MRI of the upper abdominal organ is difficult because of the large field of view and respiratory movement. GRASP is a recently developed technique that allows for the acquisition of the DCE-MRI with good spatial and temporal resolutions over a large field of view [13,14,17]. The advancement in technology has enabled the acquisition of MRI images in very short intervals with free-breathing. Free-breathing DCE-MRI is useful in patients with limited respiratory reserves. In the current study, we used free-breathing DCE-MRI, regardless of the patients’ respiratory reserve, and evaluated the perfusion characteristics of the pancreatic parenchyma. We conducted this study to investigate the possibility that multiphase MRI of the pancreas, the most often used CE MRI, was not being captured at the predicted phase.

In this study, the peak-enhancement time was longer and the peak concentration was higher in patients with pancreatic cancer than in those without pancreatic cancer. The common radiological findings of pancreatic cancer include pancreatic duct dilatation and parenchymal atrophy [18]. A decrease in pancreatic enhancement distal to pancreatic cancer has been commonly noted in previous studies [19,20]. We evaluated the relationship between pancreatic duct diameter and delay time, which corresponded to the interval between the peak-enhancement times in the aorta and pancreas. In the pancreas head and body, the delay time significantly increased as the pancreatic duct diameter increased. The results imply that blood supply in a pancreas with a dilated duct is slower than in a normal pancreas. It also suggests that a prolonged delay time may manifest as a reduction in enhancement in images acquired at a fixed time delay. Meanwhile, the peak concentration in the pancreatic parenchyma was significantly correlated to the pancreatic duct diameter. Because of the higher peak concentration and longer delay time, more contrast material may be retained in the pancreas despite the slower pace. As a result, the pancreatic phase with a fixed time delay cannot represent the pancreas with maximal enhancement and the delayed phase with a fixed time delay cannot represent the pancreas with contrast material washed out in patients with pancreatic duct dilatation. In these patients, it may change the relative signal intensity of the tumor to the pancreas parenchyma.

The time–intensity curves in the aorta, celiac axis, and SMA were also evaluated in this study, and the delay time in the pancreas could be evaluated. Several previous studies used the test bolus technique to overcome the limitations of the fixed time delay or bolus tracking techniques [7,8]. When a bolus tracking technique is used for CT or MRI, the aorta density/intensity is the reference for the scan time for the pancreas. Therefore, our results suggest that the bolus tracking technique may be inaccurate in some patients with a prolonged delay time, thus indicating the benefit of using DCE-MRI. The pancreas signal intensity is measured using the test bolus technique, and hemodynamic changes in the pancreas are fully reflected by this technique. Nevertheless, additional scan time and a split dose of contrast material are necessary for the test bolus technique. The acceptable image quality obtained with the free-breathing DCE-MRI method in this study demonstrated the feasibility of applying DCE-MRI to abdominal organs.

We evaluated the image quality of DCE-MRI, and none of the examinations were nondiagnostic. Two reviewers gave a score of two for two MRI examinations (2.7%) in terms of the overall image quality. Although pancreatic edge sharpness was the item rated with the lowest score, all of the image quality analysis items obtained acceptable scores. Previous studies also showed that GRASP DCE-MRI could achieve comparable or better image quality than conventional breath-hold contrast-enhanced MRI [14,21,22]. Additionally, streaking artifacts in GRASP images were not so prominent in this study. Because streaking artifacts occur at the peripheral portion of the body, they may not affect the pancreas, which is located at the center of the body. Additionally, a good score for respiratory artifacts is promising, even for the free-breathing image acquisition of abdominal organs. As a result, the DCE-MRI will be able to well demonstrate the contrast enhancement pattern of lesions and organs, especially when fine details of anatomic structure are not a primary focus of CE T1WI. It may also be a viable alternative to conventional contrast enhancement images in patients who have difficulties holding their breath [23].

Several limitations were observed in this study. First, a pathologic diagnosis was not available for all patients and biopsy was not performed on patients without a focal lesion on MRI, and even in some patients with pancreatic focal lesions. Second, we could not perform a radiology–histology correlation to evaluate the histologic change in the pancreatic parenchyma that leads to perfusion change on the MRI. Although some patients underwent a pancreatectomy, the number was small, and it was impossible to match the pathology slide section to the MRI. Third, we did not evaluate the diagnostic performance of the DCE-MRI. As the DCE-MRI is used to evaluate the enhancement pattern of the pancreas focal lesion, not to detect the focal lesion, we thought that determining the diagnostic performance of the DCE-MRI for detecting a pancreas focal lesion was not necessary in this study. Fourth, other imaging modalities that could validate pancreatic perfusion changes were not available. Perfusion CT can attain perfusion characteristics, although it has been limited in use owing to radiation exposure.

In conclusion, the DCE-MRI could present perfusion changes in a pancreas with pancreatic cancer and the correlation between the perfusion and morphologic changes in the pancreas. There is a possibility that images obtained at a fixed delay time may not adequately reflect the expected contrast enhancement of pancreatic parenchyma in the pancreas with ductal dilation.

## Figures and Tables

**Table 1:** MRI sequences and parameters.

	Heavily T2WI	T2WI	DWI	DCE-MRI	Delayed CE T1WI
Planes	Axial, coronal	Axial	Axial	Axial	Coronal, sagittal
Sequence	HASTE	TSE	EPI	GRASP	CAIPIRINHA-VIBE
TR (msec)	900	2520	7100	3	3.7
TE (msec)	130	95	48	1.58	1.23
Flip angle (degree)	135	120	90	9	10
Thickness (mm)	4	4	4.5	3	2.5
Interslice gap (mm)	1	1	0.9	0	0
Resolution (mm^2^)	0.7 × 0.7	1.2 × 1.2	1.6 × 1.6	1.4 × 1.4	0.8 × 0.8
Field of view (mm^2^)	400 × 325	400 × 400	400 × 320	400 × 400	300 × 400
NEX	1	1	1	1	1
B values (s/mm^2^)	-	-	0, 50, 400, 800, 1000	-	-
Acquisition time (min:sec)	0:42	1:36	4:49	5:48	0:14

TR, repetition time; TE, echo time; NEX, number of excitations; T2WI, T2-weighted imaging; T1WI, T1-weighted imaging; DCE, dynamic contrast-enhanced; DWI, diffusion-weighted imaging; CE T1WI, contrast-enhanced T1-weighted imaging; HASTE, half-Fourier acquisition single-shot turbo spin echo; TSE, turbo spin echo; EPI, echo planar imaging; GRASP, golden-angle radial sparse parallel MRI; CAIPIRINHA, controlled aliasing in parallel imaging results in higher acceleration; VIBE, volumetric interpolated breath-hold examination.

**Table 2:** Characteristics of patients.

Patient Characteristics	Value (%)
Age (years old)	71.5 ± 12.6
Sex	
Male	31 (41.3)
Female	44 (58.7)
Focal lesion	
Presence	38 (50.7)
Absence	37 (49.3)
Radiological diagnosis of focal lesion (n = 38)	
Pancreatic ductal adenocarcinoma	22 (57.9)
Cyst or cystadenoma	14 (36.8)
Solid pseudopapillary tumor	1 (2.6)
Neuroendocrine tumor	1 (2.6)
Histological diagnosis of focal lesion (n = 22)	
Pancreatic ductal adenocarcinoma	18 (81.8)
Serous cystadenoma	2 (9.1)
Intraductal papillary mucinous neoplasm	1 (4.5)
IgG4-related disease	1 (4.5)

**Table 3:** Qualitative image quality analysis of dynamic contrast-enhanced images.

	Reader 1	Reader 2	ICC
Pancreas edge sharpness	4.01 ± 0.67	3.80 ± 0.68	0.775 (0.644–0.858)
Respiratory motion artifact	4.68 ± 0.55	4.25 ± 0.72	0.462 (0.148–0.660)
Streaking artifact	4.23 ± 0.69	4.01 ± 0.76	0.716 (0.550–0.820)
Noise	4.25 ± 0.66	4.00 ± 0.68	0.679 (0.491–0.797)
Overall image quality	4.13 ± 0.78	3.93 ± 0.76	0.793 (0.672–0.869)

ICC, intraclass correlation coefficient.

**Table 4:** Peak-enhancement time and concentration in the vessels and pancreatic parenchyma.

		Value	*p*-Value		
Peak-enhancement time (s)	Vessels				
	Aorta	58.4 ± 9.0	0.917	-	0.054
	SMA	58.5 ± 8.7		0.054	-
	Celiac axis	59.5 ± 9.6	-		
	Pancreatic parenchyma				
	Head	83.2 ± 41.7	0.231	-	0.972
	Body	86.5 ± 42.7		0.512	-
	Tail	84.1 ± 36.5	-		
Peak concentration	Vessels				
	Aorta	1.08 ± 0.54	0.806	-	0.004
	SMA	1.08 ± 0.52		0.006	-
	Celiac axis	0.95 ± 0.47	-		
	Pancreatic parenchyma				
	Head	0.21 ± 0.13	0.791	-	0.728
	Body	0.21 ± 0.13		0.818	-
	Tail	0.21 ± 0.13	-		

**Table 5:** Differences in enhancement parameters according to presence of pancreatic cancer.

	Pancreatic Cancer (n = 22)	No Pancreatic Cancer (n = 53)	Benign Lesion (n = 16)	*p*-Value, Cancer vs. No Cancer	*p*-Value, Cancer vs. Benign
Peak-enhancement time (s)					
Head	102.6 ± 59.0	76.2 ± 30.1	91.1 ± 61.6	0.083	0.566
Body	108.9 ± 56.4	77.2 ± 31.8	91.9 ± 61.6	0.020	0.241
Tail	98.9 ± 28.4	77.9 ± 37.9	80.1 ± 19.7	0.013	0.018
Delay time (s)					
Head	54.2 ± 58.9	17.6 ± 27.0	23.0 ± 46.7	0.044	0.287
Body	51.1 ± 56.0	18.5 ± 28.5	21.6 ± 46.5	0.001	0.097
Tail	40.7 ± 29.0	19.6 ± 35.6	13.0 ± 19.8	0.001	<0.004
Peak concentration					
Head	0.27 ± 0.19	0.18 ± 0.08	0.20 ± 0.10	0.071	0.151
Body	0.31 ± 0.15	0.18 ± 0.08	0.18 ± 0.07	0.015	0.001
Tail	0.33 ± 0.15	0.18 ± 0.09	0.16 ± 0.06	0.019	<0.001

## Data Availability

The data presented in this study are available upon request from the corresponding author. The data are not publicly available owing to privacy or ethical concerns.
